# Evidence-Informed Deliberative Processes for Health Benefit Package Design – Part II: A Practical Guide

**DOI:** 10.34172/ijhpm.2021.159

**Published:** 2021-11-10

**Authors:** Wija Oortwijn, Maarten Jansen, Rob Baltussen

**Affiliations:** Department for Health Evidence, Radboud Institute for Health Sciences, Radboud University Medical Center, Nijmegen, The Netherlands.

**Keywords:** Health Technology Assessment, Evidence-informed Deliberative Processes, Legitimacy, Health Benefit Package

## Abstract

**Background:** Countries around the world are using health technology assessment (HTA) for health benefit package design. Evidence-informed deliberative processes (EDPs) are a practical and stepwise approach to enhance legitimate health benefit package design based on deliberation between stakeholders to identify, reflect and learn about the meaning and importance of values, informed by evidence on these values. This paper reports on the development of practical guidance on EDPs, while the conceptual framework of EDPs is described in a companion paper.

**Methods:** The first guide on EDPs (2019) is further developed based on academic knowledge exchange, surveying 27 HTA bodies and 66 experts around the globe, and the implementation of EDPs in several countries. We present the revised steps of EDPs and how selected HTA bodies (in Australia, Brazil, Canada, France, Germany, Scotland, Thailand and the United Kingdom) organize key issues of legitimacy in their processes. This is based on a review of literature via PubMed and HTA bodies’ websites.

**Results:** HTA bodies around the globe vary considerable in how they address legitimacy (stakeholder involvement ideally through participation with deliberation; evidence-informed evaluation; transparency; and appeal) in their processes. While there is increased attention for improving legitimacy in decision-making processes, we found that the selected HTA bodies are still lacking or just starting to develop activities in this area. We provide recommendations on how HTA bodies can improve on this.

**Conclusion:** The design and implementation of EDPs is in its infancy. We call for a systematic analysis of experiences of a variety of countries, from which general principles on EDPs might subsequently be inferred.

## Background

**Key Messages**

** Implications for policy makers**

- Evidence-informed deliberative processes (EDPs) provide a practical stepwise approach for health technology assessment (HTA) bodies to improve the legitimacy of their decision-making processes.
- HTA bodies can improve legitimacy through implementation of four elements in its processes: stakeholder involvement, ideally through participation with deliberation; evidence-informed evaluation; transparency; and appeal.
- We show practical examples of the application of the four elements by HTA bodies around the globe. These examples can inspire HTA practices globally.

** Implications for the public**

Health technology assessment (HTA) is used to inform decision-making, including decisions about which health technologies should (not) be (partly) reimbursed. Relevant stakeholders, eg, specific population groups who bear the consequences of these decisions such as patients and the public are often not involved in this process. However, decision-makers are increasingly urged to organise fair, legitimate processes in health benefit package design, with legitimacy referring to the reasonableness of decisions as perceived by stakeholders, including patients and the public. Evidence-informed deliberative processes (EDPs) were developed in response to this and provide a practical approach on how to improve legitimacy in decision-making processes. This paper provides insight in how HTA bodies can best address legitimacy in their processes, and how this is currently being implemented in different countries around the globe.

Countries around the world are rethinking the design of their health benefit packages to achieve universal health coverage.^1-3^ Many countries have established health technology assessment (HTA) bodies which support governments in these choices.^4^ HTA determines the value of a health technology and can inform decisions at different levels, eg, reimbursement decisions on a single health technology or regarding larger parts of the benefit package.

 Increasingly, decision-makers are urged to organise fair, legitimate processes in health benefit package design, with legitimacy referring to the reasonableness of decisions as perceived by stakeholders.^5,6^ Evidence-informed deliberative processes (EDPs) were developed in response to this.^7^ An EDP is a practical and stepwise approach for HTA bodies to enhance legitimate health benefit package design based on deliberation between stakeholders to identify, reflect and learn about the meaning and importance of values, informed by evidence on these values. In EDPs, the concept of legitimacy is translated into four elements: stakeholder involvement ideally operationalised through stakeholder participation with deliberation; evidence-informed evaluation; transparency; and appeal. The underlying idea is that HTA bodies integrate these elements into the various steps of their processes (Figure).^8^ It is hereby important to recognise that the EDP-framework is a holistic approach to enhance legitimacy, even though its terminology may suggest a narrow focus on deliberation.

**Figure:**
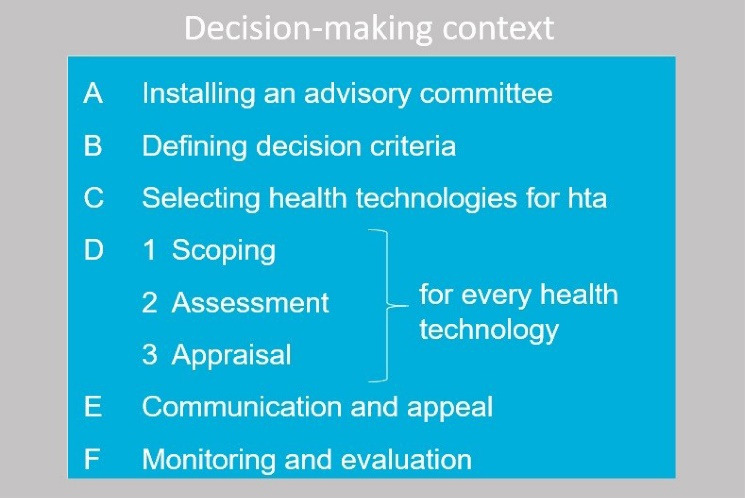
Six Steps of Implementing Evidence-Informed Deliberative Processes

 There are large differences among HTA bodies in how they address legitimacy of health benefit package design. HTA bodies sometimes organise stakeholder involvement for certain processes, eg, the Scottish Medicines Consortium and the National Institute for Health and Care Excellence (NICE) in the United Kingdom, and the National Committee for Health Technology Incorporation in Brazil routinely consult patient groups and the public as part of their HTA processes.^9^ However, this still does not mean that stakeholders are actively engaged in deliberations and can openly exchange views on argumentation and evidence. In other countries, stakeholder involvement is only in its infancies or not organised at all. Similar observations can be made for the other elements of legitimacy (evidence-informed evaluation; transparency; and appeal) – ie, HTA bodies vary widely in how they incorporate these into the various steps of their processes.^10^

 This paper reports on the further development of practical guidance on EDPs to support HTA bodies on how they can best address legitimacy in the various steps of their processes. The first EDP guide was developed in 2019^11^; the updated and comprehensive guide is released at the HTAi meeting in June 2021 and available elsewhere.^9^ The guide provides practical recommendations on how a country can improve its HTA process, taking into account that each country has a unique decision-making context and should make its own choices as to what is appropriate As such, it is not meant as a blueprint, but as an inspirational and practical tool. EDPs are currently employed by national health authorities in Ghana, Iran, Moldova, Pakistan and Ukraine for revision of their health benefit packages,^12^ and its principles were previously applied for similar use in Kazakhstan, Thailand,^13^ the Netherlands^14^ and Indonesia.^15^ A companion paper reports on the development of the conceptual framework of EDPs.^8^

 This paper first describes the development process of practical guidance on EDPs. Subsequently, for each step of the EDP framework, we provide specific guidance on key issues of legitimacy and present a novel overview on how eight relatively well-developed HTA bodies around the world (in Australia, Brazil, Canada, France, Germany, Scotland, Thailand and the United Kingdom) have made choices regarding these issues. We conclude with several overall recommendations. We speak of ‘HTA’ when referring to the whole process, while ‘hta’ specifically refers to the evaluation of a single technology.

###  Development of Practical Guidance 

 In his commentary on EDPs, Culyer describes the development of deliberative processes: “the understanding how best to make arrangements (...) immediately takes one to a highly complex academic and professional crossroads of behavioural science, governance, political philosophy, political science, the law, administrative theory, industrial economics and communications. This lattice of disciplines and professions militates against there being any single unifying ‘theory of deliberative processes’ so one needs to add other requirement: imagination and descriptive evidence. The design and execution of deliberative processes requires imaginative work by people well-grounded in the practical realities of their own culture and politics and a systematic accretion of descriptive material from which, over time, one may be able to infer some general principle.”^16,17^

 Reflecting this perspective, the development of EDPs and its operationalisation into practical guidance has indeed been a process of ‘learning by doing,’ while deepening its theoretical foundation. The overall concept of EDPs stems from the general principle of legitimacy, the definition of four elements is a practical translation of the Accountability for Reasonableness framework,^5^ the definition of practical steps is based on existing HTA methods and tools, whereas related recommendations on best practices are inferred from observed practices of HTA bodies around the world. The development process itself was geared through academic knowledge exchange^15,18-28^ and the experience of implementing EDPs in several countries. We also surveyed HTA bodies and experts around the globe on their need for guidance.^26,28^ The latter activity revealed a strong need for support on most steps of the EDP process. For example, 64% of surveyed INAHTA (International Network of Agencies for Health Technology Assessment) members and 84% of surveyed low- and middle-income country experts express a need for guidance on Step D3 Appraisal (Table 1).

**Table 1:** Need for Guidance With Respect to the Steps of EDPs as Expressed by INAHTA Members (n = 27) and Health Technology Assessment Experts in Low- and Middle-Income Countries (n = 66)^a^

	**INAHTA Members**	**LMIC Experts**
A. Installing an advisory committee	46%	70%
B. Defining decision criteria^b^	n/a	n/a
C. Selecting health technologies for hta	73%	85%
D1. Scoping	65%	81%
D2. Assessment	32%	82%
D3. Appraisal	64%	84%
E. Communication and appeal	52%	80%
F. Monitoring and evaluation	56%	86%

Abbreviations: INAHTA: International Network of Agencies for Health Technology Assessment; LMIC, low- and middle-income country. ^a^Percentages refer to the element most in need of guidance, per step. ^b^Step B has not separately been addressed in the surveys.

 The guide is organised around the steps of EDPs and addresses a total of 58 questions that HTA bodies may have on how they can best improve the legitimacy of their processes (see Box 1).^29^ The next section summarises the recommendations.

**Box 1. List of 58 Questions Addressed in the Evidence-Informed Deliberative Process Guide^9^**

** Introduction**

Why is HTA important to achieve universal health coverage?

Why a guide to enhance legitimate decision-making?

How is this guide different from other guides for benefit package design?

Why do HTA bodies need EDPs?

Whom is this guide for?

How should this guide be used?

What is different in this second version of the guide?

Can I get support to implement EDPs in my country?

** Evidence-Informed Deliberative Processes**

Why use an EDP?

What is an EDP?

What are the practical steps?

Why is stakeholder involvement important in EDPs?

What is stakeholder participation?

What is stakeholder consultation?

What is stakeholder communication?

** Context**

Why is institutional design important and how should it be assessed?

Why is policy context important and how should it be assessed?

What is current HTA capacity and how should it be assessed?

** Step A. Installing an Advisory Committee**

What is the role of an advisory committee?

What should the composition of an advisory committee be?

How can stakeholders get involved in the advisory committee?

How should the members of an advisory committee be identified and selected?

Should an advisory committee be supported by sub-committees?

What is the role of the chair of the advisory committee?

Should the advisory committee use a structured decision-making process?

How should a decision be reached?

How should undue influences in the process be avoided?

Should committee meetings be public?

Should committee members and other stakeholders be trained?

Should committee members be financially compensated?

** Step B. Selecting Decision Criteria**

Why are decision criteria needed?

What are generic decision criteria?

What are contextual decision criteria?

How should decision criteria be selected?

** Step C. Selecting Health Technologies for HTA**

What approaches are available for identifying and selecting technologies for hta?

Which approach is best for identifying and selecting technologies for hta?

Which approach should be used for choosing to identify and select health technologies for hta for disinvestment decisions?

** Step D1. Scoping**

What is scoping?

Who should be involved in scoping?

How should scoping be conducted?

** Step D2. Assessment**

What is assessment?

Who does the assessment?

How should hta findings from another setting be adapted?

** Step D3. Appraisal**

What is appraisal?

What is the aim and end product of appraisal?

Should appraisal use an explicit framework to trade-off criteria?

How is deliberation best organised?

How is evidence best presented in the appraisal step?

How much time does the advisory committee need for appraisal?

How can be avoided that an advisory committee says ‘yes’ to all technologies?

How can the advisory committee trade off the three dimensions of the UHC cube?

How should a decision be reached?

How can all argumentation in an advisory committee be best registered?

** Step E. Communication and Appeal**

How should the outcome of the deliberation of the advisory committee be communicated?

How should a formal mechanism for reviewing decisions and addressing disagreements be organised?

** Step F. Monitoring and Evaluation**

What is M&E?

Why is M&E important?

How should M&E be organised?

---------------

Abbreviations: HTA, health technology assessment; EDP, evidence-informed deliberative process; M&E, Monitoring and Evaluation.

In addition, for each EDP step, we considered the relevance of the four elements of legitimacy and selected a total of 27 key issues of legitimacy based on their importance and data availability. For example, for step A of the EDP process ‘Installing an advisory committee,’ we selected seven key issues: Mandate of the advisory committee; Accessibility of meetings; Number of members; Composition; Term; Selection of members; Reporting of conflict of interest to become member. We subsequently analysed how the eight selected HTA bodies around the globe organise the legitimacy of their processes based on these key issues. To that aim, we reviewed the (grey) literature via PubMed covering the last 2 years (ie, since the publication date of the first guide in 2019) and HTA bodies’ websites during January-February 2021. This information can be used as inspiration for other HTA bodies. The findings are presented in full in our guide and summarised in the section below (for reasons of space, we only provide a table for step A ‘Installing an advisory committee,’ tables for all EDP steps can be found in Supplementary file 1: Tables S1-S6).

###  EDPs Practical Steps: Recommendations and HTA Practices Around the Globe

####  Step A Installing an Advisory Committee 

####  Role

 We advise HTA bodies to install an advisory committee which main task it is to prepare advisory or binding recommendations on the public reimbursement and pricing of health technologies.^4^ Our analysis shows that the recommendations made by six HTA bodies are advisory (Brazil, France, Thailand, Canada, Scotland, Australia) and recommendations by two HTA bodies are binding (Germany, UK) (Table 2). The committee may be involved in other steps of the HTA process such as the selection of decision criteria (step B) or scoping (step D1). In all its tasks, the committee needs to make social and scientific judgements. We recommend HTA bodies to use deliberation to achieve this: it facilitates the judgement process and aims to create a more coherent and mutual understanding of preferences of recommendations among committee members.^30^ All analysed HTA bodies use deliberation in their processes.

**Table 2:** Key Issues of Legitimacy in Step A of the Health Technology Assessment Process

**Indicator**	**Brazil **	**France **	**Germany **	**Thailand **	**Canada **	**UK**	**Scotland**	**Australia**
**HTA Body** ^*^	CONITEC	HAS	IQWiG	HITAP	CADTH	NICE	SMC	PBAC
**Evaluated committee**	Plenary	TC	Federal Joint Committee (‘Plenum’)	Subcommittee on Determination of Types and Coverage of Health Services	CDEC	Technology Appraisals Committee	As above	As above
*Mandate of the advisory committee*	Advisory	Advisory	Binding	Advisory	Advisory	Binding	Advisory	Advisory
*Accessibility of meetings*	Open	Closed, although anyone can attend if approved by the chair	As a rule, resolutions are passed in public sessions. Closed sessions or written voting is permissible only in clearly defined exceptions	Closed	Closed	Mixed: meetings are held in public, but the agenda is divided into two parts if the committee needs to discuss confidential information	Mixed: meetings are open to the public, but occasionally, parts of the discussions may legally require a closed session to maintain the academic and commercial confidentiality	Closed. Representatives from patient groups PBAC can participate by invitation only
*Number of * *members*	13 members with voting rights	29 members with voting rights	13 members with voting rights	Not identified	16 members with voting rights	24 members with voting rights	23 members with voting rights	20 members with voting rights
*Composition*	Members from different departments of the Ministry of Health (7), National Health Agency, National Health Surveillance Agency, National Board of Health, National Council of State Health Secretaries, National Council of Municipal Health Secretaries and the Federal Board of Medicine	One chair, two vice-chairs, 20 health practitioners, one methodologist, one epidemiologist, two patients and two consumer representatives	One chair, two impartial members, members of Health Insurance Funds (5), Hospital Federation (2), Association of Statutory Health Insurance Physicians (2), Association of Statutory Health Insurance Dentists (1)	Representatives of the 3 major public health insurance funds, health professionals, financial expert, traditional medicine expert, health system research institute, civic society, the chair of the working group of topic selection	One chair, three patient representatives, one ethicist, 11 experts who represent a variety of qualifications and expertise; members are expected to have experience and knowledge related to HTA, reimbursement policy and/or epidemiology	One chair; members represent the NHS, the public, academia and industry	Members include clinicians, pharmacists, NHS board representatives, the pharmaceutical industry and the public	Members include doctors, health professionals, health economists and consumer representatives
*Term*	No term specified	Three-year term, renewable twice	Six-year term	Four-year term	Three-year term	Three-year term	Not identified	Four-year term
*Selection of members*	Closed: appointed by stakeholder organisations	Closed: appointed by HAS	Closed: appointed by stakeholder organisations	Closed: appointed by National Health Security Board	Open procedure: members are selected through a public call for nominations and appointed by CADTH	Open procedure	Open procedure	Not identified: members are appointed by the Minister for Health
*Reporting of conflict of interest to become member*	Yes	Yes	Yes	Not identified	Yes	Yes	Yes	Yes

Abbreviations: HTA, health technology assessment; HAS, Haute Autorité de Santé; IQWiG, Institute for Quality and Efficiency in Health Care; CONITEC, National Committee for Health Technology Incorporation; HITAP, Health Intervention and Technology Assessment Program; CADTH, Canadian Agency for Drugs and Technologies in Health; NICE, National Institute for Health and Care Excellence; PBAC, Pharmaceutical Benefits Advisory Committee; TC, Transparency Committee; CDEC, Canadian Drug Expert Committee; NHS, National Health Service; SMC, Scottish Medicines Consortium. * The organisations listed in the table are not all formally established as HTA bodies.

####  Composition

 As the advisory committee informs public funded decision-making, its members should preferably reflect the broad public interest in its recommendations. This means that the composition of the committee should mirror the diversity of social values present in the population. We advise HTA bodies to include 10–15 formal members in the committee which in practice may consists of two types of members.^31^ The first type includes members based on their professional or scientific expertise, such as clinicians, public health experts, ethicists, economists, or epidemiologists. The second type includes members based on the interests they represent, such as patient- and/or carer-organisations or industry. Note that these latter members represent the general interests (eg, of patients and industry) and not specific interests regarding specific health technologies. All formal committee members should have voting power to have a say in the final recommendation of the advisory committee. Our analysis shows that the number of formal committee members varies between 13 (Brazil, Germany) and 29 members (France), with one HTA body not identifying its size (Thailand). Each advisory committee includes members of both types, representing expertise and specific interests respectively. Four committees include members representing the public (or consumers) (France, UK, Scotland, Australia) and two include patient representatives (France, Canada). In each committee formal members have voting power (Table 2).

####  Selection of Committee Members

 The process for identifying and selecting committee members is preferably done through a transparent approach. The advisory committee should be effectively independent and be free from undue influences. To be cognisant of this and to reduce the risk of undue influence, it is important that committee members sign a conflict-of-interest form before taking on their term *and* before every meeting.^32^ Our analysis reveals that members are selected through open procedures by three HTA bodies (Canada, Scotland, UK). Furthermore, seven HTA bodies require advisory committee candidates to report any conflict of interest to become a member, while we could not identify this for Thailand. One committee holds open meetings (Brazil), three committees hold part of their discussions behind closed doors when required (Germany, UK, Scotland), and four committees hold closed meetings (France, Thailand, Canada, Australia) (Table 2).

####  Alternative Ways of Involving Stakeholders 

 HTA bodies may wish to involve stakeholders beyond formal membership of the advisory committee and generally three approaches to stakeholder involvement are distinguished.^33^ They can organise *stakeholder participation* by inviting specific stakeholders to participate in their meetings. These stakeholders are not formal members of the advisory committee and are not granted voting power. Such stakeholders typically represent interests or have specific expertise of the health technology being deliberated upon. Alternatively, stakeholders can be *consulted*; they can be involved in non-deliberative ways, such as through the provision of verbal comments at meetings or written testimonies prior to meetings. Another option is stakeholder *communication* in which stakeholders are only informed about the processes and/or decisions. Ideally stakeholders are provided training opportunities to familiarize themselves with the advisory committees’ procedures. In our analysis, we found that six advisory committees facilitate stakeholder consultation(s) to inform their appraisal (Brazil, France, Canada, UK, Scotland, Australia). For example, in the UK (NICE) clinical experts and patients can be consulted during the committee meeting to present their views. Two HTA bodies allow stakeholder participation (Germany, Thailand), eg, in Germany five patients and two representatives appointed by the Conference of Health Ministers of the German States have a discussion and petition rights on all agenda items. In addition, we found that two HTA bodies provide training opportunities for stakeholders (Brazil, UK) (Table 2).

####  Step B Selecting Decision Criteria 

 Decision criteria reflect the broad goals of a country’s health system (such as maximisation of population health, fair distribution of health and financial protection) and underlying values (such as equity, solidarity and access to good quality care).^34^ The advisory committee employs decision criteria for the assessment and subsequent appraisal of health technologies. In this way, recommendations on the inclusion or exclusion of health technologies in the health benefit package are based on social preferences.

 The process of criteria selection may involve several steps. We recommend to first conduct a review of policy documents on national health strategies to identify important social values of the county. These should be operationalised into measurable criteria. Second, a workshop with stakeholders should be organised in which they express their preferences vis-à-vis these values and corresponding criteria, possibly informed by a survey among a broader group of stakeholders. The result of this workshop should be to recommend a set of (generic) decision criteria. Third, the HTA body should ideally subject their list of decision-criteria to public scrutiny by means of a democratic process, for example, by publishing them and soliciting comments. Finally, depending on the decision-making structure, the final list of decision criteria should be formally endorsed, for example by the Ministry of Health.

 Our analysis of HTA bodies shows that decision criteria often relate to the health technology’s (comparative) health gains (eg, efficacy, effectiveness and safety) (Brazil, France, Germany, Canada, UK, Scotland, Australia), its cost-effectiveness (Brazil, Thailand, Canada, UK, Scotland, Australia), and its financial implications (eg, budget impact, cost (savings) outside the health sector) (Brazil, Thailand, Canada, UK, Australia). Less commonly used decision criteria relate to concerns about equity and moral aspects of access to health technologies (Thailand, Australia), ethical, legal and social implications (Canada), non-health factors and non-health gains (UK), predicted use in practice (Australia), the target population (France), patient affordability (Australia) and severity of the medical condition treated (Australia). We could not identify from the sources studied how the decision criteria are selected including whether stakeholders are involved in the selection (Supplementary file 1: Table S2). Previous research on linking health system values and decision criteria also found that it is difficult to retrieve this information from written sources^35^ For learning purposes it would be valuable to study the selection processes of decision criteria as used by HTA bodies in more detail using for example semi-structured interviews.

####  Step C Selection of Health Technologies for hta

 HTA bodies have limited budgets for their activities, so important choices need to be made about which health technologies are evaluated. Making such choices often involves two steps: (i) the identification of health technologies in need for hta; and (ii) among those, the selection of health technologies that are most important to evaluate.^36^ The responsible body may employ of broad array of approaches, ranging from ad-hoc requests and (closed, targeted, open) nomination procedures to horizon scanning systems. These approaches can all be characterised by the level of transparency, how proactive the HTA body is in the identification and selection of technologies for hta and which sources of information are used (only stakeholder input or also other sources).^37^

 Two of the analysed HTA bodies use an open procedure (France, Germany), one uses ad-hoc requests (Australia) and one selects health technologies using both ad-hoc requests and an open procedure (Thailand). Four HTA bodies use results from a horizon scanning system (Brazil, Canada, UK, Scotland), combined with either an open procedure (Brazil, UK), ad-hoc requests (Scotland), or both ad-hoc requests and a targeted procedure (Canada). Seven HTA bodies consult with stakeholders to inform their identification of health technologies, while we did not find information regarding the body in Australia. Subsequently, the seven HTA bodies use an explicit selection procedure to prioritise health technologies for HTA. From these, three HTA bodies consult stakeholders in the selection process (Brazil, Thailand, UK), and one HTA body allows stakeholders to participate in selection (Germany). We were not able to identify stakeholder involvement for the other HTA bodies (Supplementary file 1: Table S3).

####  Step D1 Scoping

 Scoping concerns the explicit definition of the objective and research questions of an hta. Scoping requires the systematic exploration of the relevant aspects of a health technology under evaluation from multiple perspectives (eg, patients, informal carers, health professionals, decision-makers). Scoping provides important input for the assessment of health technologies in the sense that it defines what evidence needs to be collected.^38^ We provide guidance on how to conduct scoping in our guide. We also advise HTA bodies to take up responsibility for scoping, but policy makers, Ministries of Health or external committees, in consultation with relevant stakeholders and/or experts, can also do this.

 Our analysis shows that six HTA bodies have a scoping procedure in place (Brazil, France, Germany, Thailand, Canada, UK), while we could not identify this for Australia and Scotland. Three HTA bodies consult stakeholders during scoping (Thailand, Canada, UK), and two HTA bodies allow stakeholders to participate in scoping (France, Germany), while the other three HTA bodies are not involving stakeholders in scoping (Supplementary file 1: Table S4).

####  Step D2 Assessment

 The assessment of health technologies includes various activities: systematic evidence collection on the selected decision criteria; synthesising evidence, including quality analysis; independent review of evidence and reporting findings and implications. Our guide does not provide detailed methodological guidance on these activities, as this already exists elsewhere, eg, in the EUnetHTA core model.^39^ Ideally the collection and provision of evidence is carried out by an independent party, such as an academic organisation, to avoid undue influence of any kind. The hta report should be subject to an independent review and discussed by relevant stakeholders, which may lead to revisions, before making the final hta report publicly available.

 Our analysis shows that all HTA bodies request an independent review of hta reports; seven HTA bodies consult with stakeholders to inform their assessments, except for Brazil. Also, seven HTA bodies make hta reports publicly available by publishing them on their website, except for Thailand (Supplementary file 1: Table S4).

####  Step D3 Appraisal

####  Deliberation 

 In the appraisal step, the advisory committee interprets the results of the assessment in a broader perspective and formulates a recommendation to inform decision-makers. This is an intrinsically complex and value-laden task and requires a careful judgement process for two reasons. First, appraisal involves social judgements on the importance of decision criteria, such as weighing the value of a life year gained in very young or old persons. Stakeholders have different interests and may judge the importance of criteria differently. Second, the assessment step typically results in different types of evidence (from various sources and study designs) involving varying degrees of uncertainty - and an advisory committee needs to judge the relevance of this evidence for the decision under scrutiny.^40^

 We recommend HTA bodies to use deliberation to achieve this. HTA bodies should report on these deliberations and include the argumentation underlying recommendations to ensure the consistency and transparency of recommendations and allowing stakeholders to comment on draft recommendations.

 The central challenge in these deliberations is to trade off the different decision criteria. A performance matrix can be a useful starting point – this presents the performance of a health technology on the generic decision criteria. There are different options for how advisory committees can trade off criteria.^40^ First, they can undertake *qualitative analysis* deliberating on the performance matrix using explicitly defined criteria. Second, they can employ *quantitative analysis* traditionally referred to as multi-criteria decision analysis and following several steps: (*i*) the evidence on each criterion in the performance matrix is translated into a score (eg, between 0 and 100); (*ii*) stakeholders’ preferences regarding the relative importance of criteria are measured using criterion weights; (*iii*) scores are multiplied by the relative weight of that criterion; (*iv*) the weighed scores are added together to obtain an overall value for each technology. Third, it can use *analysis with decision rules* interpreting the performance matrix using a set of simple rules that guide the advisory committee in making trade-offs between criteria, which can be quantitative or qualitative in nature. Irrespective of the specific approach, we advise HTA bodies to always include a deliberative component in its appraisal process.

 Our analysis shows that four advisory committees meet on a monthly basis (Thailand, Canada, UK, Scotland), three committees meet every two weeks (Brazil, France, Germany), and one committee meets three times a year (Australia). Meetings are scheduled for two sequential half days in Brazil, a full day in the United Kingdom, while for six HTA bodies we could not identify this information. Our analysis further shows that each HTA body posts minutes of their advisory meetings on their website, three HTA bodies (may) include video-recordings (Brazil, France, Germany); five HTA bodies allow stakeholders to comment on draft recommendations (Brazil, France, Germany, Canada, UK) and one HTA body does not allow stakeholders to comment (Scotland). For Australia and Thailand, we were not able to identify stakeholder involvement. Furthermore,our analysis shows that five HTA bodies use a qualitative approach to trade-off criteria (Brazil, France, Germany, Thailand, Canada); three HTA bodies use decision rules that guide the advisory committee in how the acceptability of a technology’s cost-effectiveness is modified by other criteria (UK, Scotland, Australia) or what to consider first (eg, are technologies safe and effective) (Australia). No HTA body is using quantitative analysis for trading off criteria (Supplementary file 1: Table S4).

####  Developing Reimbursement Decisions

 For the sake of legitimacy, reimbursement decisions are ideally be reached by consensus. However, the development of consensus is not always feasible because stakeholders may, for good reasons, continue to disagree. Also, from a theoretical perspective on legitimacy, it is not always necessary to reach a consensus. The objective of deliberation is to maximise understanding and support among involved stakeholders, realising that not all stakeholders necessarily need to agree with the decision.^5^ As such, an advisory committee can also reach a decision by majority voting in case consensus is not achievable. In our analysis two advisory committees rely on consensus as a closure mechanism (Brazil, Thailand), two committees use majority voting if necessary (UK, Australia), and the remaining committees (France, Germany, Canada, Scotland) rely on majority voting by default (Supplementary file 1: Table S4).

####  Step E Communication and Appeal 

 Communication and appeal are important features that enhance the legitimacy of decision-making by making the decision and underlying argumentation public, while the conditions of revision and enforcement establish responsiveness and accountability.^5^ Responsible health authorities – typically the Ministry of Health – should strive to ensure that reimbursement decisions are communicated to all relevant stakeholders, using a variety of channels. Our analysis shows that only five HTA bodies have communication strategies in place to inform stakeholders (France, Thailand, Canada, UK, Australia) (Supplementary file 1: Table S5).

 ‘Appeal’ refers to the need for a mechanism that gives stakeholders the possibility to apply for a revision of a decision, or by providing (new) arguments or evidence and receive a reasoned response.^5^ HTA bodies should establish a protocol for appeal, including the requirements regarding provision of new evidence and clear revision rules. It is important that the protocols for communication and appeal are explicitly documented and publicly available for reasons of transparency and legitimacy. Our analysis shows that each HTA body has an appeal mechanism in place (Supplementary file 1: Table S5).

####  Step F Monitoring and Evaluation

 Monitoring and evaluation (M&E) concern the process of systematically collecting data over time on a set of pre-defined indicators and, subsequently, using this data to judge if objectives are being achieved in line with expectations or if measures for improvement are required.^41^ Data collected as part of M&E efforts ideally informs the HTA body about any shortcomings in terms of how their processes are being implemented and/or its overall impact and why this may be so. This enables the HTA body to be responsive to new insights and correct for potential shortcomings in a timely and proactive manner by implementing measures for improvement. Over time, this can enhance the legitimacy of the process by ensuring the body’s continued responsiveness and accountability. The HTA body should ensure that a M&E plan is operational and described in a publicly available document and subject it to scrutiny by stakeholders. Our analysis shows that six HTA bodies have a general M&E mechanism in place (Brazil, France, Germany, Thailand, UK, Australia), while we could not identify this for Canada and Scotland. For four out of the eight HTA bodies we identified the involvement of stakeholders in their M&E processes (France, Germany, UK), including for how to involve patients, carers and members of the public (Scotland) (Supplementary file 1: Table S6).

## Discussion

 The implementation of EDPs to support health benefit package design in a range of countries in recent years has provided important insights. First, the structured approach of the six EDP steps is holding relevance in a wide variety of settings, eg, across differences in scope of analysis (from HIV-specific analysis in Indonesia to sector-wide analyses in Pakistan), funding of health technologies (from public funding in Kazakhstan to health insurance funding in Iran) and cultures (from Ghana in the African setting to Ukraine in an east-European setting). Also, the steps are organised in a natural sequence, and this appeared to be a convenient and intuitive order for the planning of activities in these settings. Second, across settings, advisory committee members are well placed to engage in deliberations but often have limited analytical capacity to fully understand the collected evidence.^42^ It is therefore important to attune the complexity of analytical tools to this capacity and concentrate on simple descriptions of key criteria and its effective visual presentation, instead of further development of complex constructs of multiple criteria such as eg, equity-weighed quality-adjusted life years.^42^ Third, committee members seem to have a strong intuitive preference for the use of quantitative approaches to trade-off decision criteria, traditionally referred to as multi-criteria decision analysis. However, this approach carries many methodological shortcomings and can only be used as a starting point for a deliberative process.^40,43^ Analysts should be aware of this, and secure methodological rigour in their approach.

 Our analysis of how eight HTA bodies organise key aspects of legitimacy in their HTA processes has several limitations. We provide an overview per March 1, 2021, and countries are continuously updating their processes and the information provided requires to be updated annually. Furthermore, our analysis is based on publicly available sources only, such as HTA bodies’ websites. It is well possible that HTA bodies undertake more activities than listed there and included in our overview. Moreover, the publicly available sources do not necessarily specify the quality of their organisational aspects. For example, while HTA bodies may state they communicate the minutes of the advisory committee deliberations, this may in practice simply be state only the decision to include or exclude a health technology without any further argumentation.^44^ We consider two main research areas to further support the use of EDPs for health benefit package design. First, as Culyer suggests in his commentary on our earlier publication on EDPs, the best approach to the further development of deliberative processes seems to be “to accumulate the experience of a variety of countries, preferably systematically, from which some general principles might subsequently be inferred.”^17^ At the same time, there is currently little (documented) experience of HTA bodies with several aspects of legitimacy, eg, stakeholder participation through the practical organisation of deliberation, as most HTA bodies use stakeholder consultation for involvement in the HTA process. It is therefore important to describe and analyse experiences of those (few) HTA bodies that do undertake such activities in greater detail. Also the way in which appeal mechanisms for HTA decisions are organized is not systematically studied and described. Another issue that we mentioned before is that the selection of decision criteria and their linkage to health system values is not explicitly described in literature of policy documents. This would require further, more qualitative research. In addition, insights from other disciplines (eg, political sciences) are indispensable here and should be considered. Second, the use of EDPs is claimed to improve the legitimacy of benefit package design but so far only anecdotal evidence is available. Rigorous M&E activities should provide insights in how stakeholders including the public perceive the priority setting process and related outcomes in terms of funded health technologies.

## Ethical issues

 Ethical approval was not necessary for this study as we used literature review and information from websites that are freely available in the public domain.

## Competing interests

 Authors declare that they have no competing interests.

## Authors’ contributions

 All authors have contributed equally, while supervisory roles lay with RB and WO.

## Supplementary files

Supplementary file 1 contains Tables S1-S6.
