# Supplementary material for: Evidence-Informed Deliberative Processes for Health Benefit Package Design – Part II: A Practical Guide
Source: Int J Health Policy Manag. 2021 Nov 10;11(10):2327–36. doi: 10.34172/ijhpm.2021.159 (PMC9808268; doi:10.34172/ijhpm.2021.159)
Supplement: Supplementary file 1 — contains Tables S1-S6. [file ijhpm-11-2327-s001.pdf]

**Article title:** Evidence-Informed Deliberative Processes for Health Benefit Package Design – Part II: A Practical Guide

**Journal name:** International Journal of Health Policy and Management

**Authors' information:** Wija Oortwijn*, Maarten Jansen, Rob Baltussen

Department for Health Evidence, Radboud Institute for Health Sciences, Radboud University Medical Center, Nijmegen, The Netherlands

(*Corresponding author: w.oortwijn@radboudumc.nl)

**Supplementary file 1.** Tables S1-S6

**Table S1. Key issues of legitimacy in Step A. Installing an advisory committee**

| **Indicator:** | **BRAZIL** | **FRANCE** | **GERMANY** | **THAILAND** | **CANADA** | **UK** | **SCOTLAND** | **AUSTRALIA** |
|---|---|---|---|---|---|---|---|---|
| **HTA body** | **CONITEC** | **HAS** | **IQWiG** | **HITAP** | **CADTH** | **NICE** | **SMC** | **PBAC** |
| **Evaluated committee** | Plenary | Transparency TC | Federal Joint Committee ('Plenum') | Subcommittee on Determination of Types and Coverage of Health Services | CDEC | Technology Appraisals Committee | As above | As above |
| *Mandate of the advisory committee* | Advisory | Advisory | Binding | Advisory | Advisory | Binding | Advisory | Advisory |
| *Accessibility of meetings* | Open | Closed, although anyone can attend if approved by the chair | As a rule, resolutions are passed in public sessions. Closed sessions or written voting is permissible only in clearly defined exceptions | Closed | Closed | Mixed: meetings are held in public, but the agenda is divided into two parts if the committee needs to discuss confidential information | Mixed: meetings are open to the public, but occasionally, parts of the discussions may legally require a closed session to maintain the academic and commercial confidentiality | Closed. Representatives from patient groups PBAC can participate by invitation only |
| *Number of members* | 13 members with voting rights | 29 members with voting rights | 13 members with voting rights | Not identified | 16 members with voting rights | 24 members with voting rights | 23 members with voting rights | 20 members with voting rights |

| | | | | | | | | |
|---|---|---|---|---|---|---|---|---|
| *Composition* | Members from different departments of the Ministry of Health (7), National Health Agency, National Health Surveillance Agency, National Board of Health, National Council of State Health Secretaries, National Council of Municipal Health Secretaries and the Federal Board of Medicine | One chair, two vice-chairs, 20 health practitioners, one methodologist, one epidemiologist, two patients and two consumer representatives | One chair, two impartial members, members of Health Insurance Funds (5), Hospital Federation (2), Association of Statutory Health Insurance Physicians (2), Association of Statutory Health Insurance Dentists (1) | Representatives of the 3 major public health insurance funds, health professionals, financial expert, traditional medicine expert, health system research institute, civic society, the chair of the working group of topic selection. | One chair, three patient representatives, one ethicist, 11 experts members who represent a variety of qualifications and expertise; members are expected to have experience and knowledge related to HTA, reimbursement policy and/or epidemiology | One chair; members represent the NHS, the public, academia and industry | Members include clinicians, pharmacists, NHS board representatives, the pharmaceutical industry and the public | Members include doctors, health professionals, health economists and consumer representatives |
| *Term* | No term specified | Three-year term, renewable twice | Six-year term | Four-year term | Three-year term | Three-year term | Not identified | Four-year term |
| *Selection of members* | Closed: appointed by stakeholder organisations | Closed: appointed by HAS | Closed: appointed by stakeholder organisations. | Closed: appointed by National Health Security Board | Open procedure: members are selected through a public call for nominations and appointed by CADTH | Open procedure | Open procedure | Not identified: members are appointed by the Minister for Health |
| *Reporting of conflict of interest to become member* | Yes | Yes | Yes | Not identified | Yes | Yes | Yes | Yes |

Abbreviations used in the Tables:

HTA, health technology assessment;

HAS, Haute Autorité de Santé;

IQWiG, Institute for Quality and Efficiency in Health Care;

CONITEC, National Committee for Health Technology Incorporation;

HITAP, Health Intervention and Technology Assessment Program;

CADTH, Canadian Agency for Drugs and Technologies in Health;

NICE, National Institute for Health and Care Excellence;

SMC, Scottish Medicines Consortium,

PBAC, Pharmaceutical Benefits Advisory Committee;

TC, Transparency Committee;

CDEC, Canadian Drug Expert Committee;

NHS, National Health Service;

SCBP, Subcommittee for Development of Benefit Package and Service Delivery

**Table S2. Key issues of legitimacy in step B. Selecting decision criteria**

| **Indicator:** | **BRAZIL** | **FRANCE** | **GERMANY** | **THAILAND** | **CANADA** | **UK** | **SCOTLAND** | **AUSTRALIA** |
|---|---|---|---|---|---|---|---|---|
| **HTA body** | **CONITEC** | **HAS** | **IQWiG** | **HITAP** | **CADTH** | **NICE** | **SMC** | **PBAC** |
| **Evaluated committee** | Plenary | TC | Federal Joint Committee (‘Plenum’) | Subcommittee for Development of Benefit Package and Service Delivery (SCBP) | CDEC | Technology Appraisals Committee | As above | As above |
| *Reimbursement decision criteria* | Efficacy, accuracy, effectiveness, safety, cost-effectiveness, budget impact. | Actual clinical benefit, likely clinical added value compared to treatment alternatives, target population. | Level of additional patient benefit versus the appropriate comparative therapy defined as recovery, relief from pain or discomfort, improvement in quality of life, extension of life, reduction of side effects. | Cost effectiveness as main criterion, budget impact, equity and moral aspects of access to health services. | Unmet need, efficacy, effectiveness, safety, cost-effectiveness, budget impact, may include ethical, legal and social implications. | Clinical effectiveness and health-related factors, cost-effectiveness, non-health factors (social value judgments), cost (savings) outside NHS and non-health gains. Additional criteria for end-of-life medicines. | Clinical effectiveness and cost-effectiveness. | Comparative health gain (effectiveness and safety), comparative cost-effectiveness, other relevant factors (e.g. patient affordability, predicted use in practice and financial implications, equity and severity of the medical condition treated). |
| *Stakeholder involvement* | Not identified | Not identified | Not identified | Not identified | Not identified | Not identified | Not identified | Not identified |

**Table S3. Key issues of legitimacy in step C. Selection of health technologies for hta**

| **Indicator:** | **BRAZIL** | **FRANCE** | **GERMANY** | **THAILAND** | **CANADA** | **UK** | **SCOTLAND** | **AUSTRALIA** |
|---|---|---|---|---|---|---|---|---|
| **HTA body** | **CONITEC** | **HAS** | **IQWiG** | **HITAP** | **CADTH** | **NICE** | **SMC** | **PBAC** |
| **Evaluated committee** | Plenary | TC | Federal Joint Committee ('Plenum') | SCBP | CDEC | Technology Appraisals Committee | As above | As above |
| *Health technology identification procedure* | Horizon scanning; open procedure | Open procedure by the Ministry of Health | Open procedure with annual submissions | Open procedure with annual submissions (non-drugs); ad-hoc requests (drugs) | Horizon scanning; targeted procedure; ad-hoc requests | Horizon scanning; open procedure | Horizon scanning; ad-hoc requests | Ad-hoc requests |
| *Health technology selection procedure(s) to prioritise for hta* | Explicit | Explicit | Explicit. | Explicit | Explicit | Explicit | Explicit | Not identified |
| *Stakeholder involvement in the identification of health technologies* | Yes, with consultation. Stakeholders can submit technologies for assessment. | Yes, with consultation. Patient and/or carer organisations may identify health technologies. | Yes, with consultation. Industry and providers can submit dossiers and the public can provide topics. | Yes, with consultation. The working group on topic nomination, consisting of seven stakeholder groups, identifies non-drug technologies for hta. | Yes, with consultation. Interested individuals or entities can submit topics for consideration. | Yes, with consultation. Clinicians, patients and the public, and other organisations, such as the NHS, can also identify potential topics. | Yes, with consultation. SMC consults a network of clinical experts to support their horizon-scanning function. | Not identified |
| *Stakeholder involvement in the selection of health technologies* | Yes, with consultation – internal and external review with expert involvement. | Not identified | Yes, with participation. IQWiG selects up to five topics based on 15 preselected topics by a committee of public members, patients and one representative of the Commissioner for Patients' Affairs. | Yes, with consultation. The Working group on Topic Selection, consisting of four stakeholder groups, selects non-drug technologies for hta. | No. Final decision about HTA topics undertaken by CADTH will be done quarterly and is based on a ranked list, the resource needs for the topics, and CADTH's capacity. | Yes, with consultation – internal and external review with expert involvement. | Not identified | Not identified |

**Table S4. Key issues of legitimacy in steps D1-3. Scoping, assessment, appraisal**

| **Indicator:** | **BRAZIL** | **FRANCE** | **GERMANY** | **THAILAND** | **CANADA** | **UK** | **SCOTLAND** | **AUSTRALIA** |
|---|---|---|---|---|---|---|---|---|
| **HTA body** | **CONITEC** | **HAS** | **IQWiG** | **HITAP** | **CADTH** | **NICE** | **SMC** | **PBAC** |
| **Evaluated committee** | Plenary | TC | Federal Joint Committee ('Plenum') | SCBP | CDEC | Technology Appraisals Committee | As above | As above |
| **Scoping (D1)** | | | | | | | | |
| *Scoping procedure* | Yes | Yes | Yes | Yes | Yes | Yes | Not identified | Not identified |
| *Stakeholder involvement in scoping of health technologies* | No, although industry request private scoping meetings prior to submission. | Yes, with participation of experts and stakeholders. | Yes, with participation. Selected patient representatives are involved in the determination of an appropriate comparator therapy. | Yes, with consultation. Experts, health practitioners and key stakeholders are invited to comment on the scope of the research in a stakeholder meeting. | Yes, with consultation. Input from stakeholders, such as patient groups, clinical experts, drug programs and expert committee members, is considered. | Yes, with consultation. Patients, carers and stakeholders are invited to comment on the draft scope in a written consultation. An oral consultation may follow. | No | No |
| **Assessment (D2)** | | | | | | | | |
| *Publicly available assessment reports on website* | Yes | Yes | Yes | Some reports are publicly accessed in through publications, at the remit of researchers. | Yes | Yes | Yes | Yes |
| *Stakeholder involvement in the assessment process* | No | Yes, with consultation. External experts may be appointed as rapporteurs, invited to present their reports and answer questions. | Yes, with consultation. Patients and clinicians are consulted. | Yes, with consultation | Yes, with consultation. Patient and clinician group input is summarised in a clinical report. | Yes, with consultation | Yes, with consultation. If the recommendation is negative, the manufacturer can request a Patient and Clinician Engagement (PACE) meeting to gather further information on the added value of a medicine. | Yes, with consultation. PBAC can request stakeholder meetings (e.g. with manufacturers, patient groups and medical specialists) to gather additional information |
| *Independent review of the* | Yes, with consultation of | Yes, with consultation of | Yes, with consultation. | Yes, with consultation. | Yes, with consultation. | Yes, with consultation by | Yes, with consultation of new drugs | Yes, with consultation |

| | | | | | | | | |
|---|---|---|---|---|---|---|---|---|
| *evidence* | external contractors. | experts (from health professional organisations and patient or user associations). | Industry, federations and experts can submit statements on the results HTA report within a three-week time period after publication. | Experts, health practitioners and key stakeholders are invited to comment on HTA report. Final HTA report is subject to external peer review. | After review reports are finalised by CADTH, reports are sent to expert review committees. | Evidence Review Groups or Assessment Groups. | committee and testimonies from clinical experts. | |
| **Appraisal (D3)** | | | | | | | | |
| *Approach to trade-off criteria* | Qualitative | Qualitative | Qualitative | Qualitative | Qualitative | Decision rules (‘structured decision-making’). Above an ICER of £20,000 per QALY gained, both qualitative modifiers (e.g. the degree of uncertainty around the ICER) and quantitative modifiers (e.g. end-of-life treatment) can be considered. | Decision rules. Qualitative modifiers of cost-effectiveness (e.g. whether it concerns rare diseases or the absence of other therapeutic options) can be considered. | Decision rules using steps: i) are technologies safe and effective (comparative health gain)?; ii) are they cost-effective? Qualitative modifiers of cost-effectiveness are: rule of rescue, unmet needs and equity; iii) are there other relevant factors to consider? |
| *Closure mechanism* | Consensus, or majority vote if necessary. | Majority vote. At least 12 voting members need to be present. Chair has casting vote in case of equal division of votes. | Majority vote. The committee passes a resolution if at least seven votes have been cast in its favour. | Consensus | Majority vote | Consensus, or majority vote if necessary. Before a decision to vote is made, the chair will consider whether continuing the discussion at a subsequent meeting is likely to lead to consensus. | Majority vote | Consensus, or majority vote if necessary. |
| *Public record of deliberation* | Yes: minutes and video | Yes: minutes are available on | Yes: minutes and video recording are | Yes: minutes are available on | Yes: minutes are available on | Yes: minutes are available on | Yes: minutes are available on website. | Yes: minutes are available on |

| | | | | | | | | |
|---|---|---|---|---|---|---|---|---|
| | recording are available on website. | website. Video recording may be placed on website if decided by HAS President. | available on website. | website. | website. | website. | | website. |
| *Frequency of appraisal meetings* | Every two weeks | Every two weeks | Every two weeks | Monthly | Monthly | Monthly | Monthly | Three times a year, usually in March, July and November. |
| *Duration of meetings* | Meetings last two sequential half days. | Not identified | Not identified | Not identified | Not identified | 10:00 until 17:00, unless otherwise advised. | Not identified | Not identified |
| *Stakeholder involvement beyond committee membership* | Yes, with consultation. External specialists may be invited to meetings. Patients may register to make statements at the Plenary. | Yes, with consultation. Experts may present and answer questions during the committee meetings, but will not attend deliberations and voting. | Yes, participation without voting. Five patients and two representatives appointed by the Conference of Health Ministers of the German states have a discussion and petition rights on all agenda items. There are representatives of several associations with participation rights on specific topics. | Yes, participation without voting. | Yes, with consultation of external experts, patients and caregivers. | Yes, with consultation. Clinical experts and patients can be consulted to present their views. | Yes, with consultation. | Yes, with consultation. |
| *Opportunity for stakeholders to comment on draft recommendations following committee meetings* | Yes: via public consultation within 20 days. | Yes: for manufacturer only. | Yes: for payer, patients organisations, physicians and hospital representatives. | Not identified | Yes: all draft recommendations are posted on the website for stakeholder feedback. | Yes: consultees and commentators can comment if the advisory committee does not recommend use of the technology. | No: draft recommendations are published online after meeting, but there is no opportunity to comment. | Not identified |
| *Training of stakeholders* | Yes: committee members are trained in HTA. Specific forms are also available for | Not identified | Not identified | Not identified. HITAP and other organisations provide HTA training to | Not identified, although there is a dedicated patient engagement team to coordinate and | Yes: the Public Involvement Programme (PIP) supports and develops public involvement across | Not identified. There is guidance on website and dedicated Public Involvement staff. | Not identified |

| | | | | | | | | |
|---|---|---|---|---|---|---|---|---|
| | public hearings: for technical-scientific contributions and for contributions with reports of experience or opinion of stakeholders. | | | stakeholders. | assist with patient group input. | NICE's work programme. A PIP public involvement adviser is assigned to each appraisal and supports patient and carer consultee organisations, their representatives and individual patients or carers throughout the appraisal. | | |

**Table S5. Key issues of legitimacy in step E. Communication and appeal**

| **Indicator:** | **BRAZIL** | **FRANCE** | **GERMANY** | **THAILAND** | **CANADA** | **UK** | **SCOTLAND** | **AUSTRALIA** |
|---|---|---|---|---|---|---|---|---|
| **HTA body** | **CONITEC** | **HAS** | **IQWiG** | **HITAP** | **CADTH** | **NICE** | **SMC** | **PBAC** |
| **Evaluated committee** | Plenary | TC | Federal Joint Committee ('Plenum') | SCBP | CDEC | Technology Appraisals Committee | As above | As above |
| *Communication strategy to inform stakeholders* | No, only published on website. | Yes: sent to government, sponsor and published on website. | No, published on website. | Yes: adjusted to target audience (i.e. public, health professionals, researchers and patients). Recommendations are published in journals, magazines or other media. They are also sent to discussion groups and distribution lists. | Yes: all communications for drug review programmes are consolidated into a single email newsletter issued once per week. | Yes: the communications lead is responsible for circulating and communicating the guidance to appropriate groups within the NHS in England, to patients and the public. NICE also publishes a lay-version for patients and carers (known as 'information for the public'). | No, once a decision is made, it is shared in confidence with NHS boards and the pharmaceutical company four weeks before it is published to ensure that Area Drug and Therapeutics Committees can take steps to prepare for the introduction of the new medicine in health boards. | Yes |
| *Appeal mechanism* | Yes: appeals to the secretariat's decision should be made within | Yes: industry may provide written comments or request a hearing within ten | Yes | Yes | Yes | Yes: all consultees have the opportunity to consider an appeal against the final appraisal determination and have the | Yes: industry may request a meeting following publication of a not recommended advice for a full or resubmission. | Yes |

| | | | | | | | | |
|---|---|---|---|---|---|---|---|---|
| | ten days starting from the date of publication in the official magazine. If appeals are accepted, there are hearings with the public. | days of receipt of the draft recommendation. | | | | opportunity to report factual errors. | | |

**Table S6. Key issues of legitimacy in step F. Monitoring and evaluation**

| **Indicator:** | **BRAZIL** | **FRANCE** | **GERMANY** | **THAILAND** | **CANADA** | **UK** | **SCOTLAND** | **AUSTRALIA** |
|---|---|---|---|---|---|---|---|---|
| **HTA body** | **CONITEC** | **HAS** | **IQWiG** | **HITAP** | **CADTH** | **NICE** | **SMC** | **PBAC** |
| **Evaluated committee** | Plenary | TC | Federal Joint Committee (‘Plenum’) | SCBP | CDEC | Technology Appraisals Committee | As above | As above |
| *Mechanism for monitoring and evaluation* | Yes, at the request of Ministry of Health or related organisations. | Yes, if requested by stakeholders, ministry of health or HAS. Otherwise, every five years. | Yes | Yes | Not identified | Yes | Not identified | Yes: specific medicines are monitored (e.g. through post-market reviews of Pharmaceutical Benefits Scheme Subsidised Medicines). |
| *Stakeholder involvement in monitoring and evaluation* | Not identified | Yes, with consultation | Yes, with consultation | Not identified | Not identified | Yes | Yes, with participation. The Public Involvement Network (PIN) is made up of patient and carer groups who have submitted evidence to SMC. PIN also has a core advisory group which works with SMC to continuously improve how they involve patients, carers and members of the public in their work. | Not identified |
